# Hair cell force generation does not amplify or tune vibrations within the chicken basilar papilla

**DOI:** 10.1038/ncomms13133

**Published:** 2016-10-31

**Authors:** Anping Xia, Xiaofang Liu, Patrick D. Raphael, Brian E. Applegate, John S. Oghalai

**Affiliations:** 1 Department of Otolaryngology-Head and Neck Surgery, Stanford University, 801 Welch Road, Stanford, California 94305, USA; 2 Department of Anorectal Surgery, the First Affiliated hospital of China Medical University, 155 NanjingBei Street, ShenYang, LiaoNing Province 110001, China; 3 Department of Biomedical Engineering, Texas A&M University, 5059 Emerging Technology Building, 3120 TAMU, College Station, Texas 77843, USA

## Abstract

Frequency tuning within the auditory papilla of most non-mammalian species is electrical, deriving from ion-channel resonance within their sensory hair cells. In contrast, tuning within the mammalian cochlea is mechanical, stemming from active mechanisms within outer hair cells that amplify the basilar membrane travelling wave. Interestingly, hair cells in the avian basilar papilla demonstrate both electrical resonance and force-generation, making it unclear which mechanism creates sharp frequency tuning. Here, we measured sound-induced vibrations within the apical half of the chicken basilar papilla *in vivo* and found broadly-tuned travelling waves that were not amplified. However, distortion products were found in live but not dead chickens. These findings support the idea that avian hair cells do produce force, but that their effects on vibration are small and do not sharpen tuning. Therefore, frequency tuning within the apical avian basilar papilla is not mechanical, and likely derives from hair cell electrical resonance.

## Abstract (web summary)

The avian auditory papilla has many similarities to the mammalian cochlea but whether force generation by hair cells amplifies the travelling wave, as it does in mammals, remains unknown. Here the authors show that the chicken basilar papilla does not have a ‘cochlear amplifier' and that sharp frequency tuning does not derive from mechanical vibrations.

The inner ear transduces the mechanical energy of sound into electrochemical signals that are encoded within the auditory nerve. This originates when sound-induced pressure waves deflect stereociliary bundles at the apical poles of sensory hair cells. Ionic flow through mechanoelectrical transduction channels generates receptor potentials that modulate synaptic release from the basal poles of the hair cells. However, hair cells do not only sense mechanical force: they also produce force. The stereociliary bundle can produce force that appears to be associated with opening of the transduction channel^1^,^2^ and/or fast adaptation associated with closing of the transduction channel^3^,^4^. Stereociliary bundle force production is likely present to some degree in every type of hair cell across all vertebrates and may aid frequency tuning^5^. In mammals, outer hair cells (OHCs) also produce force by changing their cell length in response to changes in their transmembrane potential, a process that requires prestin protein, termed electromotility^6^,^7^. Together, these forces produced by hair cells give rise to otoacoustic emissions, sounds that emanate from the inner ear. Otoacoustic emissions have been found in all vertebrate classes^8^.

However, there are obvious anatomical and physiological differences between the inner ears of different species that argue for substantial differences in how they transduce sound^9^. For example, within the auditory papillae of commonly studied vertebrates such as frogs, lizards and turtles, the sensory hair cells are anatomically quite similar and are clustered with minimal organization over a plane. In frogs, there is no basilar membrane (BM)^10^. In most lizards and turtles, the hair cells sit on a vibratory BM that is not mechanically tuned^11^,^12^. Also, there are non-specialized supporting cells around the hair cells that give rise to new hair cells when existing hair cells are damaged (that is, hair cell regeneration). Physiologically, frog, lizard and turtle hair cells have electrical resonance, in which the complex interplay between the voltage-gated channels tunes the receptor potential of each hair cell to a select frequency^13^,^14^,^15^,^16^. Thus, when a hair cell stereociliary bundle is stimulated at the frequency to which it is tuned, it will best stimulate the auditory nerves that receive synaptic input from it.

In contrast, the mammalian cochlea is a coiled structure in which the sensory hair cells are precisely aligned by highly-specialized supporting cells atop a vibratory BM. Along the length of the cochlea, there are three rows of OHCs and one row of inner hair cells (IHCs). Furthermore, mammalian hair cells do not regenerate. Functionally, a principle feature of the mammalian cochlea is that the passive mechanics of the BM produce a spectral analysis on the sound energy which manifests as travelling waves^17^. Sound energy is distributed over the length of the cochlea in a tonotopic manner, so that high-frequency sounds cause the BM at the base to vibrate maximally and low-frequency sounds cause the BM at the apex to vibrate maximally. The active force generation properties of OHCs are commonly thought to locally amplify and sharpen the spatial extent of the BM vibrations. This is termed cochlear amplification and it improves auditory sensitivity to quiet sounds and frequency selectivity^6^,^18^. An alternative concept is that the active properties of OHCs do not occur on a cycle-by cycle basis, but instead adjust the amount of local damping present within a non-amplifying system^19^,^20^. According to both theories, however, functional OHCs are required to achieve the normal sharpness of cochlear vibratory responses. Electrical resonance has not been found within mature mammalian hair cells. Instead, IHCs sense the frequency tuning inherent to the mechanical vibrations of the organ of Corti and convey these complex, nonlinear signals to the afferent auditory nerve^21^.

The avian basilar papilla has similarities to both the mammalian cochlea as well as the auditory papillae of other non-mammalian species^22^. Like mammals, there are two types of hair cells, short and tall. Short hair cells (SHCs) sit on a vibratory BM and are only minimally innervated by afferent neurons^23^. In contrast, tall hair cells (THCs) sit adjacent to them on a stiff fibrocartilage plate (FcP) and are heavily innervated by afferent neurons. The role of the SHCs is unknown, but they have been hypothesized to produce force that amplifies and/or sharpens the mechanical stimulus driving the stereociliary bundles of the THCs, somewhat analogous to the way that mammalian OHCs provide this function for IHCs^24^,^25^. Chicken hair cells have both bundle motility and prestin motors that enhance bundle motility, which together can move the tectorial membrane (TM) in a radial direction^26^. Consistent with this, birds produce otoacoustic emissions in a manner consistent with hair cell force production^27^. The similarities with the mammalian cochlea are limited, however. Like frogs, turtles and lizards, bird hair cells are arrayed in a plane, are surrounded by a non-specialized supporting cells layer that supports hair cell regeneration, and have electrical tuning^28^.

Thus, the avian basilar papilla offers a way to study how passive BM mechanics, active properties of hair cells, and hair cell electrical resonance all relate to the evolution of frequency tuning and auditory sensitivity. Furthermore, as an intermediary organ between the auditory papillae of reptiles and amphibians and the cochlea of mammals, these studies may provide clues as to the functional roles of these processes in mammalian hearing. Specifically, we sought to determine whether amplification, defined as increased vibratory amplitude and sharpening of frequency tuning, occurs within the chicken basilar papilla. To accomplish this, we measured sound-induced vibrations within the apical half of the chicken basilar papilla *in vivo*. We found that, similar to mammals, there was a travelling wave. However, travelling wave vibrations were linear and not amplified. Furthermore, the frequency tuning of the travelling wave was broad and did not match the sharpness found in previously published single-unit chicken auditory nerve recordings. These data demonstrate that the chicken basilar papilla does not have a ‘cochlear amplifier' like in mammals, and suggest that sharp frequency tuning in chickens derives from hair cell electrical resonance.

## Results

### *In vivo* imaging of the chicken basilar papilla

**Figure 1:**
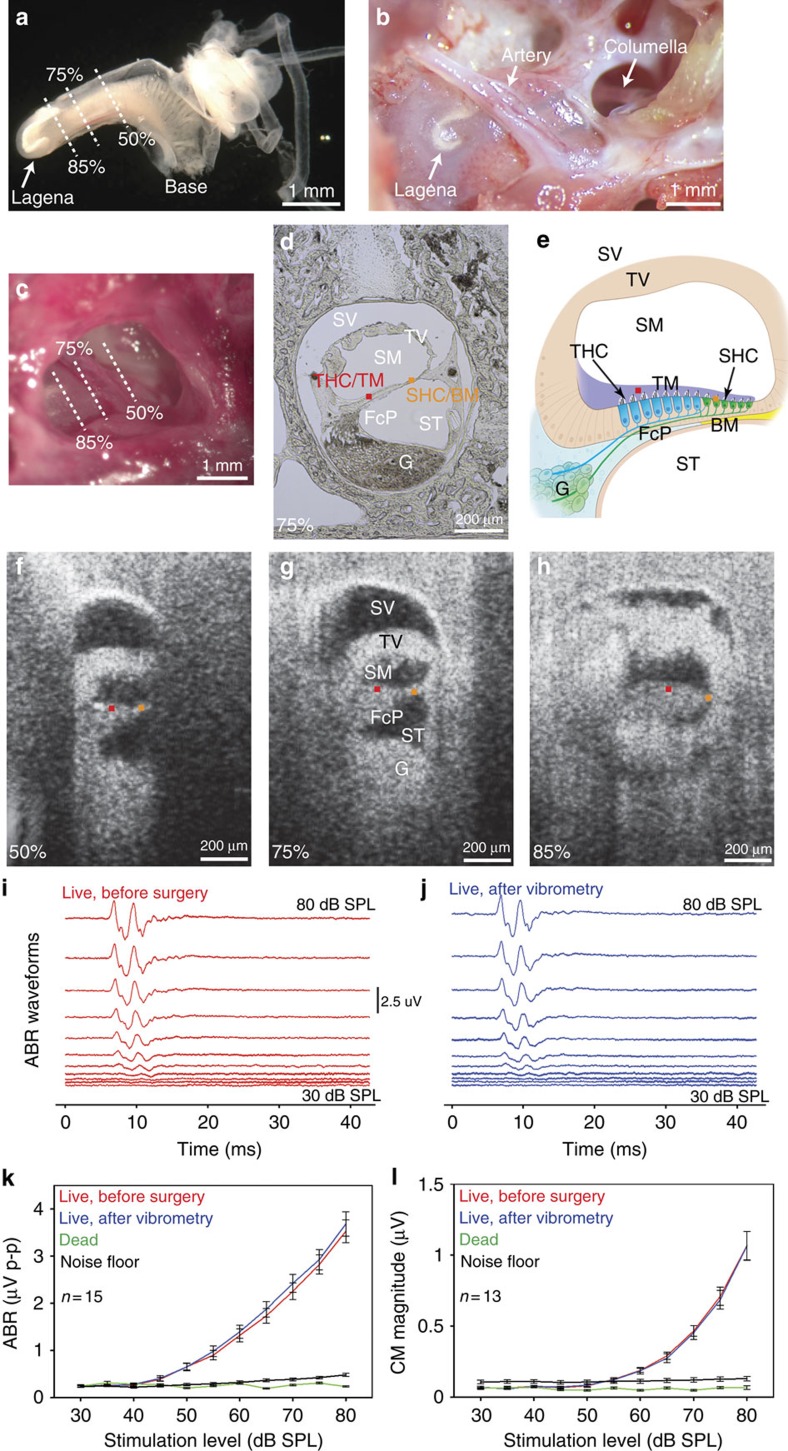
Representative experimental preparation of the P5-10 chicken basilar papilla. (**a**) Microdissected inner ear tissues in which the surrounding otic capsule bone has been removed. The semicircular canals are the tubular structures to the right. The lagena is at the apical end of the basilar papilla. The three locations along the length of the basilar papilla that we imaged *in vivo* (50, 75 and 85% from the base) are highlighted. (**b**) View after opening the middle ear bulla widely in a post-mortem chicken. This involved removing about half of the tympanic membrane. The columella that connects the tympanic membrane to the base of the basilar papilla is seen. An artery angles over basilar papilla. (**c**) *In vivo* view. (**d**) Frozen section across the basilar papilla 75% from the base. Tuning curves were measured at the SHC/BM region (orange dot) and the THC/TM region (red dot). (**e**) Illustration of the chicken basilar papilla. Scala vestibuli (SV), scala media (SM), scala tympani (ST), tegmentum vasculosum (TV), fibrocartilage plate (FcP), auditory nerve ganglion cells (G). (**f**–**h**) *In vivo* VOCTV images across the basilar papilla 50, 75 and 85% from the base. (**i**,**j**) ABR waveforms in one representative live chicken immediately after induction of anaesthesia, before surgery and repeated again after surgery to open the middle ear bulla and performing vibrometry experiments. There was no change in waveform morphology. The stimuli were clicks of intensity from 30 to 80 dB SPL in 5 dB steps. (**k**) Peak-to-peak responses from the ABR signals as a function of stimulus intensity. (**l**) CM magnitude responses to 2.75 kHz tones that ranged in intensity from 30 to 80 dB SPL in 5 dB steps. Error bars show the s.e.m.

We studied the P5-10 chicken basilar papilla *in vivo.* At this age, auditory thresholds and auditory nerve tuning curves are mature^29^,^30^. We used volumetric optical coherence tomography and vibrometry (VOCTV) to measure sound-induced vibrations of the tissues inside the inner ear without opening the surrounding otic capsule bone^31^. We had to limit our studies to the apical half of the basilar papilla because surgically accessing more basal regions would require resecting some or all of the tympanic membrane, inhibiting sound conduction (Fig. 1a–c and Supplementary Movie 1). We recorded at locations 50, 75 and 85% from the base of the basilar papilla, which is ∼3.9 mm long^32^.

For localization, we compared the cross-sectional *in vivo* images obtained with our VOCTV system to *ex vivo* histologically frozen sections (Fig. 1d–h). While the spatial resolution of our imaging system prevented the identification of single cells, key anatomic regions could be localized. The three fluid-filled scalae (vestibuli, media and tympani) were obvious. The tegmentum vasculosum, analogous to the stria vascularis in the mammalian cochlea because it maintains the electrochemical gradients within the endolymph, was clearly visible as a thick structure separating scala vestibuli from scala media. However smaller structures, such as the SHCs, THCs, the TM, the BM and the FcP under the THCs, could not be discretely identified. Nevertheless, the anatomic image was adequate to localize two important regions: the SHC/BM region and the THC/TM region. We selected the centres of these regions and measured their vibration patterns in response to sound stimuli.

### Physiological assessment of auditory function

Even though the vibratory measurements we made were performed without creating an opening into the inside of the basilar papilla, it is still possible that auditory function was in some way compromised in the experimental preparation. Therefore, we assessed the health of the peripheral auditory system by making electrophysiological recordings using auditory brainstem responses (ABRs) and cochlear microphonics (CMs). ABRs provide a comprehensive assessment of the function of the middle ear, basilar papilla and auditory neurons, whereas CMs provide an assessment of the summed receptor potential that stem from hair cell transduction^33^. ABRs were measured using click stimuli, similar to previously published recordings^34^,^35^,^36^. CMs were measured using 2.75 kHz sine wave stimuli. In a subgroup of chickens, we recorded ABRs and CMs at three time points during the experiment. The first time was just after induction of anaesthesia (that is, before any of the surgical procedures were started), the second time was after the vibratory measurements were finished in the living chicken, and the third time was after euthanasia but before the vibratory measurements were repeated in the dead chicken.

ABR waveforms showed no change in morphology after the vibratory measurements were completed in the living chickens compared with the baseline recordings (Fig. 1i,j). Similarly, there was difference in the peak-to-peak amplitudes between the two measurements (Fig. 1k) (two-way analysis of variance (ANOVA), *P*=0.30; *n*=15). Auditory thresholds, defined as the stimulus intensity at which the peak-to-peak ABR amplitude rose to 5x above the average noise floor measured during the last 20 ms of the recordings, were not different after completion of the vibrometry compared with baseline (41.3±1.9 versus 42.2±2.0 dB SPL, mean±s.e.m.; paired *t*-test, *P*=0.59; *n*=15). These thresholds are slightly lower than has been previously published (∼50 dB SPL)^36^, but importantly, they remained stable throughout the experiment. Post-mortem, our data confirmed that there were no ABR waveforms and that the peak-to-peak responses dropped to within the noise floor.

Similarly, the CM magnitude demonstrated no changes between the baseline recordings and the recordings made after performing vibrometry in the living chickens (Fig. 1l) (two-way ANOVA, *P*=0.84; *n*=13). The CM also disappeared post-mortem. Together these data demonstrate that our experimental preparation was viable and argue that peripheral auditory function during vibrometry studies in living chickens was intact. Furthermore, these data confirm that euthanasia produces the expected loss of auditory function. In particular, the drop in the CM to the point where they were undetectable supports the concept that there was a large drop in hair cell transduction currents in our experimental preparation after killing.

### Mechanics of the SHC/BM region

**Figure 2:**
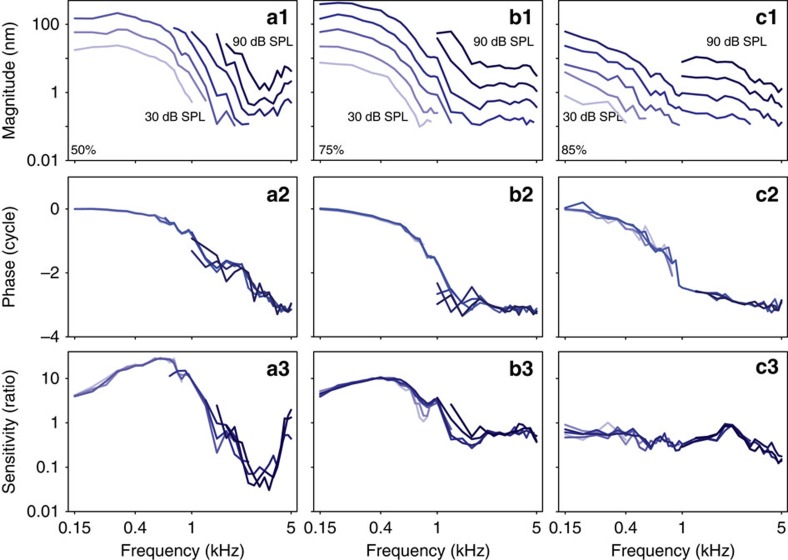
Representative vibratory responses of the SHC/BM region. (**a**) 50% from the base. (**b**) 75% from the base. (**c**) 85% from the base. (Top) The vibratory magnitude. Magnitude responses at high sound levels and at low frequencies were not shown due to saturation of our system (explained in Methods). (Middle) The phase normalized to columella phase. (Bottom) The sensitivity was the magnitude of BM vibration divided by the vibratory magnitude of the columella.

With the laser of our VOCTV system oriented perpendicular to the BM, we measured the transverse motion of the SHC/BM region in response to pure tone stimuli. The magnitude responses demonstrated low-pass filter characteristics (Fig. 2, top row). The corner frequency shifted to lower frequencies as the measurement location was moved apically. Increasing the stimulus intensity led to proportionally larger vibratory displacements, a linear response. The phase responses (Fig. 2, middle row) followed the magnitude responses, demonstrating a phase lag that increased as the stimulus frequency was increased. The lack of nonlinearity in the magnitude responses was highlighted by normalizing the vibration of the SHC region to the vibration of the columella (Fig. 2, bottom row). The response curves all overlapped. Interestingly, these normalized data demonstrated that the basilar papilla, independent of the middle ear, had tuned responses. This was because the transfer function of the chick middle ear had low-pass filter characteristics with a very low corner frequency (<150 Hz; discussed further below).

**Figure 3:**
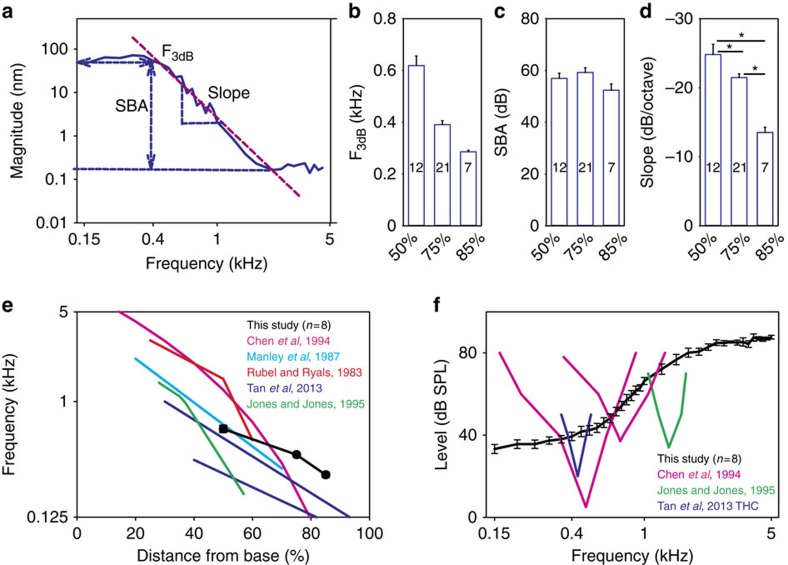
Mechanical responses do not match hair cell and auditory nerve responses. (**a**) The vibratory responses were fit with a low-pass filter model to calculate the corner frequency (*F*_3db,_**b**), the stop-band attenuation (SBA, **c**), and the filter slope (slope, **d**). (**e**) The tonotopic map we estimate using the corner frequencies was similar to previously published maps of the chicken basilar papilla. (**f**) The sound level needed to produce a 30 nm magnitude vibration at the 75% location was not tuned, unlike the threshold plots from previously published measurements in auditory nerve fibres and hair cells. Error bars show the s.e.m. Asterisks in **d** represent statistical significance *P<0.05.

However, the important point is that the net mechanical response of the chick peripheral auditory system, reflecting the mechanical stimulus seen by the hair cells, is low-pass in nature. Therefore, we fit each data set with a low-pass filter model to permit quantitative comparisons (Fig. 3a). From the vibratory responses measured in each chicken to a 60 dB SPL stimulus over the frequency spectrum, we calculated the *F*_3dB_, the stop-band attenuation, and the slope of the roll-off. The *F*_3dB_ of the mechanical responses we measured was similar to tonotopic maps of the chicken basilar papilla derived from single-unit auditory nerve studies^29^,^37^,^38^, patch-clamped hair cells recordings of electrical resonance^39^,^40^, and patterns of hair cell loss after acoustic trauma^32^,^41^,^42^ (Fig. 3b,e). On the basis of our data, the formula for the chicken tonotopic map using a logarithmic fit was:


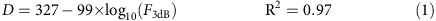


where *D* is the distance from the basal end of the basilar papilla (in %) and *F*_3dB_ is the corner frequency of the mechanical vibratory response (in Hz). The stop band attenuation was similar at the three different locations (one-way ANOVA, *P*=0.13) whereas the roll-off slope was lower at more apical locations (one-way ANOVA followed by non-paired *t*-tests, *P*_50%-75%_=0.017, *P*_50%-85%_ <0.001 and *P*_75%-85%_<0.01)(Fig. 3c,d).

While the mechanical vibratory pattern of chicken basilar papilla demonstrated a tonotopic distribution, the low-pass filter characteristics of the mechanical responses did not match the band-pass filter characteristics found within auditory neurons or hair cells. We calculated vibratory threshold plots at the location 75% from the base by determining the sound intensity required to evoke a 30 nm magnitude displacement (Fig. 3f). When compared against representative auditory nerve single-unit tuning curves^37^,^43^ or patch-clamped hair cell recordings^39^, it is clear that the mechanical response of the chicken BM was not tuned in a similar fashion. Thus, the transverse vibration of the BM cannot be solely responsible for the sharp frequency tuning found within the auditory nerve. This contrasts with the mammalian cochlea, where the tuning in the BM vibratory response is nearly identical to the tuning of the auditory nerve^21^.

### The basilar papilla supports travelling wave propagation

The existence of travelling waves within the avian inner ear remains unresolved. On one hand, point measurements of basilar papilla vibration measured in pigeons demonstrate frequency-dependent phase lags^44^ and recordings from brainstem neurons in the chicken brainstem demonstrate phase/frequency relations^45^ that are both consistent with travelling waves. On the other hand, auditory neuronal recordings in the barn owl indicate that the derived group delays do not match the tonotopic spatial frequency representation of the basilar papilla^46^,^47^,^48^.

**Figure 4:**
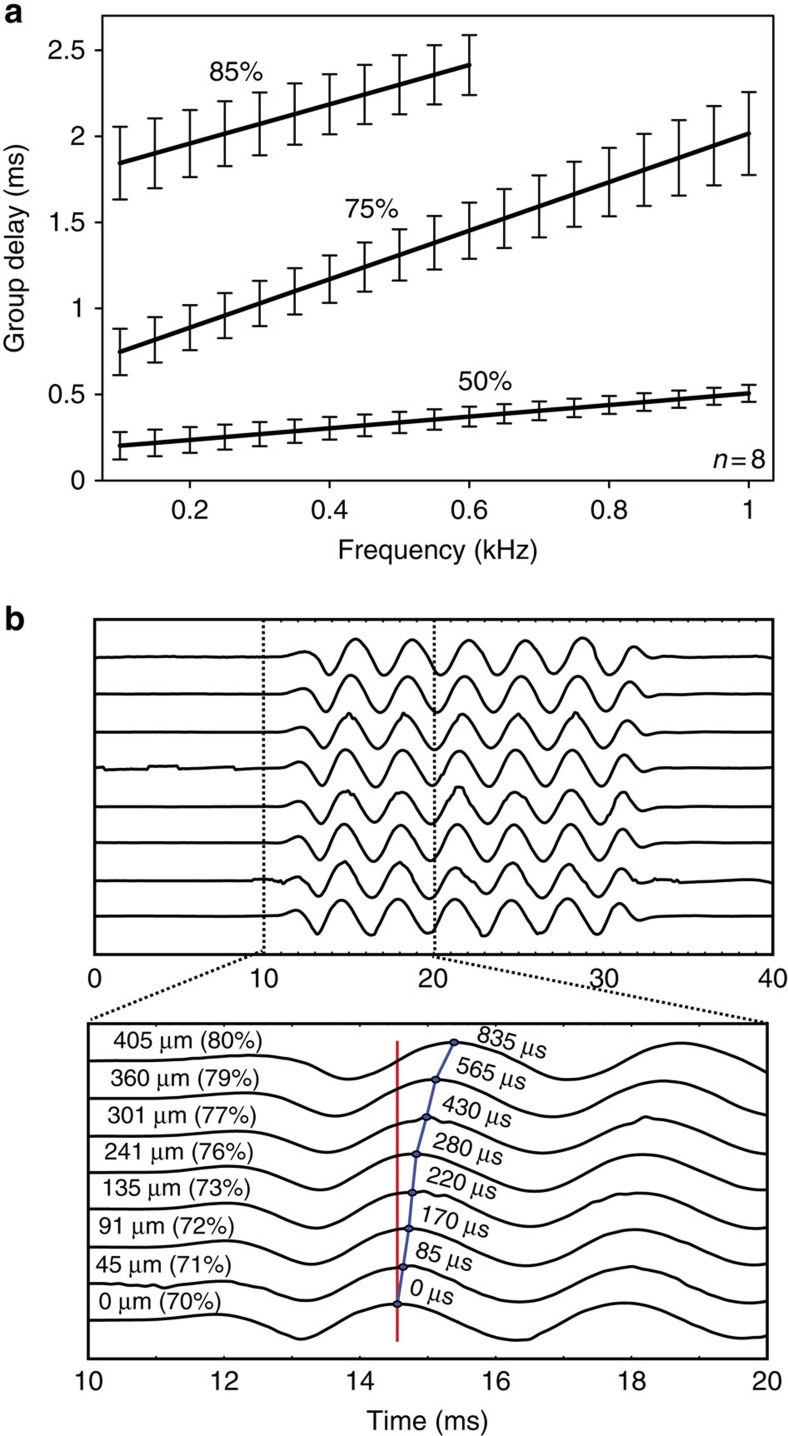
Group delay demonstrates the propagation of travelling waves. (**a**) Representative group delay calculated from the phase curves from a cohort of eight chickens. The group delay was larger for more apical locations and for higher-frequency stimuli. Error bars show the s.e.m. (**b**) Vibration measurements for eight different locations along the length of the basilar papilla in another representative chicken. A 300 Hz stimulus was presented and the time differences were determined between the first vibratory peak at each location (blue line) referenced to the basal-most recording site (red line). All magnitudes were scaled to be identical to make it easier to assess for time delays, so the *y* axis is unlabelled. It took longer for more apical sites to begin vibrating, thus demonstrating delays associated with travelling wave propagation. The distance from the basal-most recording location, the percent distance from the base, and the time delay are provided for each measurement.

A critical feature of a travelling wave is that it takes time for the vibration created by a given sound stimulus to propagate from the base to the apex, thus the group delay should be larger at more apical locations^17^,^49^,^50^,^51^. Because of the tonotopic layout of the basilar papilla, high-frequency stimuli should also have larger group delays than low-frequency stimuli. Therefore, we used the vibratory data measured at the 50, 75 and 85% distances from the base in eight chickens to derive the group delay. This was done by calculating the instantaneous slope of the phase versus frequency plots by fitting them with quadratic functions and taking the derivatives. We found larger group delays at the apical locations, and for all locations, higher-frequency stimuli had larger group delays (Fig. 4a), consistent with travelling wave propagation.

To confirm this conclusion, we then performed a different experiment where 300 Hz tone pip stimuli were presented and the vibratory motion was measured at multiple locations along the length of the basilar papilla. We measured the time delay between the first vibratory peak at eight different locations that ranged between 70 and 80% of the distance from the base. As would be expected with travelling wave propagation, the group delay was longer for more apical measurement locations (Fig. 4b). Together, these data demonstrate that the chicken basilar papilla supports travelling wave propagation, consistent with the notion that it functions like a series of longitudinally coupled filter-banks as does the mammalian cochlea^17^.

### Radial motion does not demonstrate linear or nonlinear gain

As shown in Fig. 2, the transverse vibratory response of the chicken BM is different from that of the mammalian BM is that it does not demonstrate nonlinear gain. In the mammalian cochlea, this means that the ratio of BM vibration to sound intensity is greater for quiet stimuli than for loud stimuli. One reason why the mammalian cochlea may achieve this is because the force of prestin-based somatic electromotility is directed transversely^7^. In contrast, the prestin-based force production that has been found in chicken SHCs and THCs is directed in the radial direction through tilting of the cuticular plate^26^. Thus, it is possible that nonlinear gain in the chicken could be missed if only transverse motion is measured.

**Figure 5:**
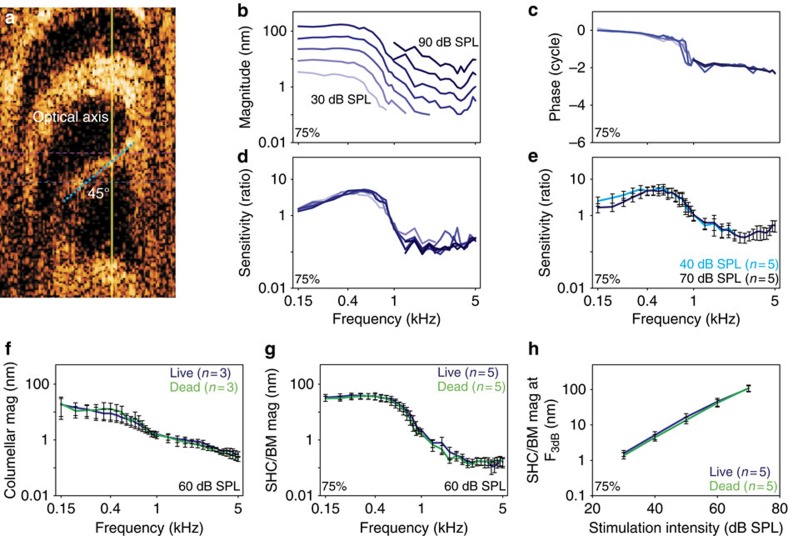
There is no detectable amplification in the transverse or radial directions. (**a**) The chicken was rotated so that the BM (blue dotted line) was oriented at a 55° angle to the optical axis of the laser in this representative example (yellow line). The vibratory magnitude (**b**), phase (**c**) and sensitivity (**d**) measured from a representative chicken. (**e**) The sensitivity of the SHC/BM region to 40 and 70 dB SPL stimuli were similar, indication a lack of nonlinear gain. (**f**) The vibratory magnitude of the columella was the same in living and dead chicken. (**g**,**h**) The vibratory magnitude of the SHC/BM region was the same in living and dead chicken, indicating a lack of linear gain. Error bars show the s.e.m.

We thus rotated the preparations to achieve an angle of 45–55° to the optical axis. This provided a measurement that substantially reflects the amount of motion in the radial direction, as determined by the vector projection on the optical axis, that is, 70.7–81.9% radial vibration (Fig. 5a). We recorded from five chickens at a location 75% from the base with this approach. Similar to the previous transverse vibration measurements, there was no evidence of nonlinear gain (Fig. 5b–d). We averaged the normalized responses at both 40 and 70 dB SPL stimuli, and these data confirmed that there were no differences in the response patterns (two-way ANOVA, *P*=0.197; Fig. 5e).

Although the mechanical response of the chicken basilar papilla does not have nonlinear gain, it is possible that linear gain might exist. In this case, there would be a proportional increase in vibrational magnitude at all stimulus intensities. To test for this possibility, we measured vibratory responses in both live and dead chickens. Care was taken to measure from the same location, either the columella or the SHC/BM region, before and 15 min after animal euthanasia. As discussed previously, the columella response demonstrated low-pass filter characteristics with a corner frequency that was below our measurement range (<150 Hz; Fig. 5f). Importantly, there were no significant differences in the vibratory magnitudes of either the columella or the SHC/BM region over the frequency spectrum or intensity range (two-way ANOVA, *P*=0.616, 0.33, and 0.88, respectively; Fig. 5f–h). Together, these data demonstrate that there was no measureable linear or nonlinear gain to pure tone stimuli in the chicken basilar papilla that were vulnerable to death. The vibratory responses appeared passive and linear.

### Mechanics of the THC/TM region

The THCs are responsible for delivering the afferent signal to the auditory neurons, but they do not sit on the vibratory BM. This raises the question of how the stereociliary bundles of the THCs are stimulated so that the chicken can hear. To address this question, we measured transverse vibratory responses at the 75% location at both the SHC/BM region and THC/TM regions in nine chickens.

**Figure 6:**
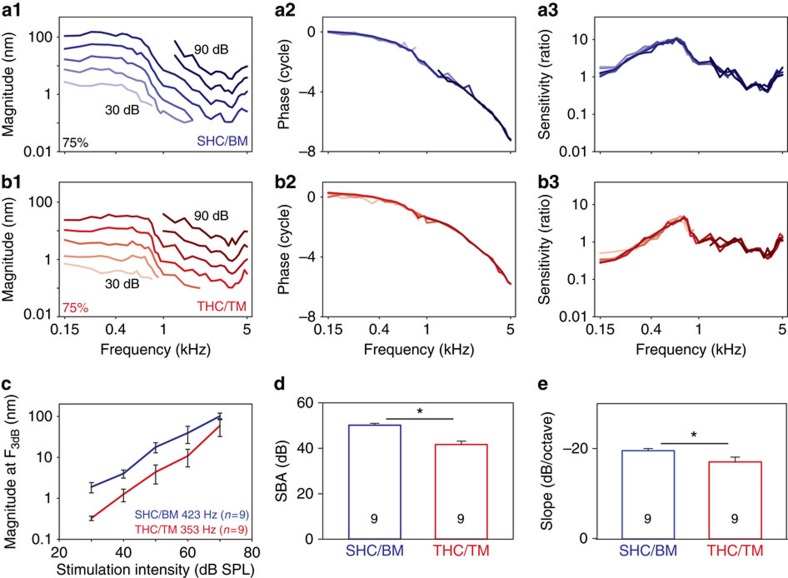
The THC/TM region vibrates in response to sound stimuli. The vibratory magnitude, phase and sensitivity from the SHC/BM region (**a**) and the THC/TM region (**b**) from one representative chicken 75% from the base. (**c**) The THC/TM region vibrated with lower magnitudes than the adjacent SHC/BM region. (**d**,**e**) The stop-band attenuation and slope were reduced in the THC/TM region compared with the SHC/BM region. Error bars show the s.e.m. Asterisks represent statistical significance *P<0.01 (**d**) and *P<0.05 (**e**).

We found that, even though it sits on the FcP, the THC/TM region vibrates substantially. It had linear characteristics, similar to the SHC/BM region but with lower magnitudes (Fig. 6a,b). For statistical comparison, the magnitude of the vibratory response at the *F*_3db_ was higher in the SHC/BM region compared with the THC/TM region (two-way ANOVA, *P*=0.018; Fig. 6c). Nevertheless, the vibratory response of the THC region was consistent with levels known to evoke mechanoelectrical transduction responses in chicken hair cells *ex vivo* and mammalian hair cells *in vivo*^31^,^39^. The stop band attenuation and the slope of the roll-off were different between the two regions (paired *t*-test, *P*<0.01 and *P*<0.05, respectively; Fig. 6d,e).

**Figure 7:**
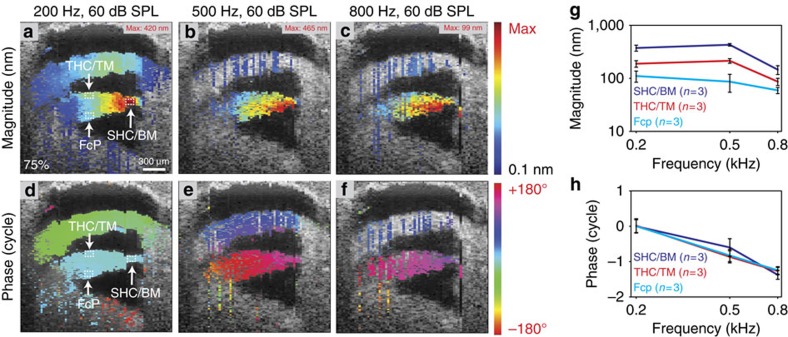
The vibratory pattern has a single mode that is centred upon the BM. (**a**–**c**) Representative vibratory magnitudes across the basilar papilla 75% from the base to 200, 500 and 800 Hz, 60 dB SPL stimuli. The magnitude is plotted in pseudocolour with the maximum vibration given in the upper right corner of each image. The largest vibration magnitudes occurred at the SHC/BM region, but the FcP and the THC/TM region also demonstrated vibration. (**d**–**f**) Vibratory phases to the same stimuli in the same chicken plotted in pseudocolour. There was little variation across the sensory epithelium, indicating a single vibratory mode was present. (**g**) Quantification of the magnitude demonstrated differences between the three different regions. (**h**) There was no phase variation between the three regions. Error bars show the s.e.m.

We then performed vibrational measurements over a cross-section of the basilar papilla at a location 75% of the way from the base (Fig. 7). We presented a constant acoustic stimulus at 200, 500 or 800 Hz at 60 dB while the laser was stepped across the specimen. The magnitudes and phases of the vibratory responses were displayed using pseudocolour-gradient scales. Data are presented for every voxel in which the vibratory response was above the noise floor of 1 nm. The magnitude responses were normalized to the maximum vibratory response in the image. The phase of the region of the BM at 200 Hz was zeroed, and all other phase data were referenced to this. The largest magnitude of vibration was centred on the BM. Importantly, all structures within the sensory epithelium, including the BM, TM, SHCs, THCs and FcP vibrated together in phase at all frequencies.

To quantify the data contained within these images, we averaged the magnitude and phase of vibration within 10 pixel boxes at three different regions (SHC/BM, THC/TM and the FcP) from three chickens. For all frequencies, the largest vibration magnitudes occurred at the SHC/BM region. Away from this area, the magnitudes were reduced (ANOVA followed by non-paired *t*-tests, *P*<0.01 for all comparisons). Nevertheless, the FcP and THC/TM regions demonstrated vibratory responses typically considered adequate to support sound transduction (that is, magnitude >1 nm). Furthermore, this proves that the FCP is not a rigid structure at acoustic frequencies.

For consistency, the phase data measured with this approach were incremented by one cycle at 500 Hz and was incremented by two cycles at 800 Hz, in order to match the progressive phase lag we found when phase was measured with smaller frequency steps (for example, Figs 2b2, 4c and 5a2). At every frequency, all three regions vibrated in phase (two-way ANOVA, *P*>0.05). Therefore, there is a single vibratory mode of the chicken basilar papilla which clearly contrasts with the frequency-dependent, multi-modal vibratory patterns found within the mammalian organ of Corti^31^,^52^,^53^.

### Labile distortion products are present

One aspect of bird auditory physiology that requires reconciliation is the generation of otoacoustic emissions, which supports the concept of hair cell force generation within the intact organ. However, unlike in the mammalian cochlea, we could not detect any evidence of travelling wave amplification in the chicken basilar papilla when a single frequency stimulus was presented. To look for evidence of active properties within the chicken basilar papilla that might be too subtle to detect with single-tone stimuli, we studied the creation of distortion products. When tones of two different frequencies are presented, nonlinearities of the mechanoelectrical transduction process produce distortion products. Active properties associated with hair cells can amplify these distortion products so that they can be measured both in the vibratory response of the BM and as distortion product otoacoustic emissions (DPOAEs). Since there is no stimulus tone at the same frequency, distortion products are relatively easy to detect with Fourier analysis.

**Figure 8:**
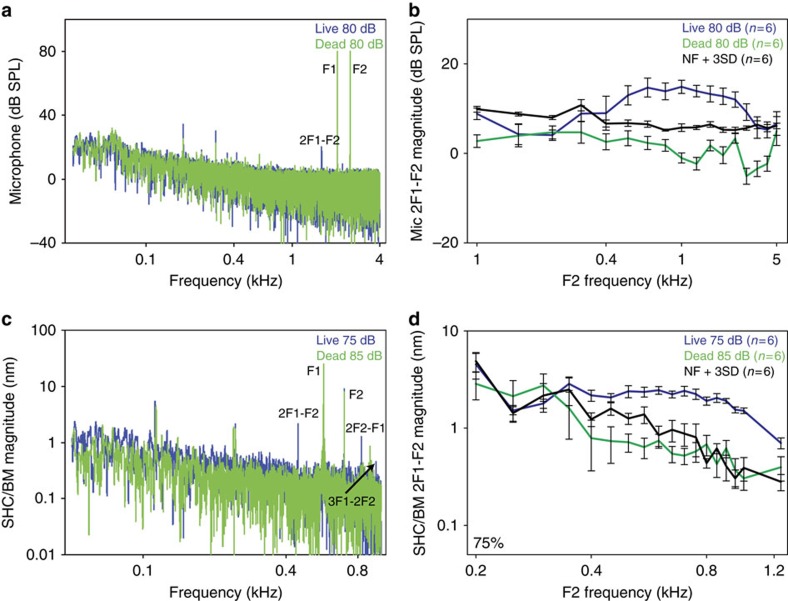
Distortion products emanate from the sensory epithelium. (**a**) A representative Fourier transform of a recording from a microphone near the tympanic membrane from a chicken in response to two pure tones (F1, F2). In the live animal, a DPOAE could be detected (2F1-F2). This was not found post-mortem. The other peaks in the frequency spectrum were caused by speaker distortions and were present in both the living and post-mortem conditions. (**b**) 2F1-F2 DPOAEs were found in live but not dead chickens. (**c**) A representative Fourier transform of a vibratory recording from the SHC/BM region 75% from the base from a chicken in response to two pure tones (F1, F2). In the live animal, distortion products could be detected (2F1-F2, 2F2-F1, and 3F1-2F2) that were not found post-mortem. The other peaks in the frequency spectrum were caused by speaker distortions and were present in both the living and post-mortem conditions. (**d**) 2F1-F2 distortion products in the vibratory responses were found in live but not dead chickens. Error bars show the s.e.m.

We measured DPOAEs over the frequency spectrum of 100 Hz to 5 kHz by presenting two simultaneous pure tones F2=1.2 × F1 at equal intensities, and measuring the acoustic pressure at the 2F1-F2 frequency in the ear canal with a microphone. Simultaneously, we measured the vibratory response of the SHC/BM region at the 75% location. We found DPOAEs only within frequencies ranging from 2 to 5 kHz (Fig. 8a,b). These responses were labile, and disappeared within 15 min of euthanasia, demonstrating that they stem from the animal and not from within our stimulus or measurement setup.

The magnitude of the DPOAEs we measured were ∼65–70 dB below the stimulus intensity of the primary tones. While one publication found that this intensity difference between the DP and the primary tones was less than what we found (∼50 dB) (ref. 54), a large body of previously published DPOAE data from chickens demonstrate that this difference is similar to what we found, ranging from 60–75 dB (refs 55, 56, 57, 58, 59). Furthermore, it has been well-established that chickens generate DPOAEs that are substantially lower in magnitude than those commonly measured in many laboratory animals, even though the phase characteristics of the DPOAEs among the different species are similar^55^. Therefore, together with the ABR and CM recordings (as shown in Fig. 1), these data support the concept that our experimental preparation was healthy and that the function of the basilar papilla was not compromised.

The distortion products we measured as otoacoustic emissions were not detectable as vibrations of the SHC/BM region. However, this is not surprising because the frequencies were much higher than the corner frequency of our measurement location (∼400 Hz). Nevertheless, we found robust distortion product vibrations in the BM at lower frequencies (range 400–1,200 Hz) (Fig. 8c,d). These too were labile and disappeared after animal euthanasia. Interestingly, we could never detect these low-frequency distortion products as DPOAEs with the microphone.

Since distortion products are present in living but not dead chickens, we interpret these data to mean that an active process is present within the basilar papilla of the live chicken. This active process is strong enough to amplify distortions generated during the transduction of two simultaneously applied tones. These distortions can emanate out of the ear canal when generated in the basal end of the basilar papilla near the columella footplate, but not when generated in the apical end of the basilar papilla. However, the effect of the active process on the vibration of the basilar papilla at the sound stimulus frequency is negligible compared with the magnitude of the passive travelling wave.

## Discussion

Herein, we show that mechanical properties of the chicken peripheral auditory system do not match previously published measures of frequency tuning in chicken hair cells and auditory nerves. Rather than demonstrating sharp frequency tuning, the chicken basilar papilla vibrates with low-pass filter characteristics. Thus, low-frequency sounds produce vibrations along most of the length of the basilar papilla that are similar in amplitude, before the response rolls off near the apex (Supplementary Movies 2–4). This means that many more hair cells will be stimulated mechanically than will convey an electrical response to the auditory nerve. Our finding that mechanical tuning is much broader than previously measured electrical and neural tuning is consistent with the concept that frequency tuning in birds derives from the electrical characteristics of their hair cells and not the underlying mechanical response of the BM.

Furthermore, while active hair cell properties are present and measurable within the apical half of the chicken basilar papilla as labile distortion products, there is no evidence that there is amplification or sharpening of the travelling wave. Thus, there is no ‘cochlear amplifier', regardless of whether the mammalian cochlear amplifier involves the popular concept of the injection of power by hair cells^18^ or the alternative concept of active hair cell properties modulating local damping to control a fluid waveguide^19^. In either case, the mechanism of frequency tuning in the chicken basilar papilla is significantly different from that of the mammalian cochlea, where auditory nerve tuning matches the mechanical vibrations of the organ of Corti^21^. However, we could only study the apical half of the chicken basilar papilla, in which the *F*_3dB_ was <650 Hz. Regardless, the frequency limit of hair cell electrical resonance has been predicted to reach 4.3 kHz in the chicken, which is sufficient is provide sharp tuning over the entire frequency range of hearing^60^. Thus, sharp mechanical tuning may not be necessary in birds. Alternatively, the tuning properties and force production by stereociliary bundle mechanics can function at acoustic frequencies^2^,^61^ which may be the primary source of hair cell tuning at higher frequencies. In addition, the possibility does exist that cochlear amplification may occur within the basal region of the chicken basilar papilla, which we could not access. Consistent with this idea, the level of prestin in chicken hair cells is higher in the base than in the apex^26^. There are also significant gradients along the length of the basilar papilla with regards to the size, orientation, and numbers of stereocilia per hair cell^62^, which may affect the ability of the bundle to produce and transmit force.

There has been only one prior report, to our knowledge, in which BM vibration was measured in a living bird^44^, in which pigeons were studied using the Mossbauer technique. This work was done before modern optical interferometric techniques were used for studying cochlear mechanics, and because of the trauma associated with opening the bone of the basilar papilla and the mass of the radioactive source that had to be placed on the BM, questions have persisted regarding their finding of purely passive mechanical responses. Our measurements were made using a less invasive approach and performed in an avian species that permits comparison with the significant amount of hair cell and auditory nerve data that are available. Our data demonstrate that the travelling wave is a primarily a passive phenomenon in birds.

Furthermore, our data argue that the theories of force production by SHCs in order to transfer mechanical energy to the THCs (which convey afferent signals to the auditory nerve) are incorrect. While OHCs perform this role and transfer energy to IHCs in the mammalian cochlea through the TM^63^,^64^, this does not happen to a significant level in the apical half of the chicken basilar papilla. Consistent with this, the lack of collagen in the avian TM suggests that it would be difficult to transfer force through it^65^,^66^. In addition, the avian TM is anchored to the apical surface of the basilar papilla at the perimeter of each hair cell^67^, making it difficult to envision how forces could be directed radially through it. Instead of needing SHCs to provide the force to stimulate their bundles, our data indicate that the FcP is not a rigid structure and that it vibrates with adequate magnitude to permit the THCs to be stimulated, independent of the SHCs. Thus the role of SHCs, if any, remains unclear. One possibility is SHCs provide a way for efferent activity to modulate the stiffness of the epithelium, which is perhaps mediated via prestin. Further work in other species that have a similar inner ear anatomy (other birds or perhaps some reptiles), but in which the basal end is more accessible, may be necessary to answer this question.

## Methods

### Animal preparation

The study protocol was approved by the Stanford IACUC and all experiments conform to the relevant regulatory standards. Both male and female chicks (*Gallus domesticus*) were studied between days P5-10. Chicks were anaesthetized with ketamine and xylazine and the temperature was maintained at 40–41 °C with a heating pad. The head was fixed with hard clay. A tracheotomy was performed to secure the airway. The musculature was removed to expose the apical half of the basilar papilla without disturbing the otic capsule bone. The artery crossing the basilar papilla was carefully protected. The VOCTV measurements started from base to apex and the hearing was monitored with ABR and CMs during the experiment. After performing all the desired experiments in the living chicken, the animal was killed by anaesthesia overdose so as not to move the head. Further measurements were made post-mortem. Finally, vibration measurements were made from the columella to determine the input to the inner ear.

### Volumetric optical coherence tomography and vibrometry (VOCTV)

The VOCTV system was custom-built and has been fully described previously^31^. It comprised a broadband swept-source with a centre wavelength of 1,310 nm and 50 kHz sweep rate (SSOCT-1,310, Axsun Technologies, Billerica, MA), dual-balanced photodetector (WL-BPD600MA, Wieserlabs, Munich, Germany), and digitizer (NI-5761, National Instruments, Austin, TX). An adaptor attached to the bottom of a dissecting microscope (Stemi-2000, Zeiss, Jena, Germany) scanned the beam in both the *x* and *y* directions. For all experiments, the power on the sample was 16 mW. The spatial resolution of our system was measured to be 9.8 μm laterally, assessed by visually discriminating separate lines on an Air Force target, and 15 μm axially in air, assessed as the full-width, half-maximum reflection from a mirror. When imaging through the otic capsule bone *in vivo*, the spatial resolution is degraded, and is estimated to be 20–25 μm in each dimension^68^.

Sound stimuli were synthesized in software and output by speakers (MDR EX37B, Sony, Tokyo, Japan) connected to an ear bar that were inserted into ear canal. We calibrated the stimuli and measured DPOAEs using a probe-tip microphone in the ear bar as previously described^69^. At any given *x*-*y* location of the scan mirror, the vibratory data from all pixels along the optical path of the laser were collected and analysed simultaneously. Our most common stimulus protocol was to present tones between 0.1 and 1 kHz in frequency steps of 0.05 kHz at intensities between 30 and 70 dB SPL in 10 dB steps, and between 1 and 5 kHz in frequency steps of 0.25 kHz at intensities between 50 and 90 dB SPL in 10 dB steps. This stimulus protocol was selected so that we did not waste time collecting vibratory data that was likely to be below the noise floor (0.1–0.2 nm under typical experimental conditions) or above the saturation point (500–2,000 nm depending upon the software settings) of our system.

The duration of the sound stimulus ranged from 50 to 200 ms. When possible, this was adjusted to achieve a noise floor that was at least 20 dB below the vibration magnitude or 0.1 nm, whichever was greater. Thus, lower sound intensities required longer measurement times. In addition, vibration data from any given voxel were not analysed if the anatomic image intensity of that voxel was <3 s.d. of the noise floor of the background intensity or if the vibration magnitude at the stimulus frequency was below a threshold set at the mean+3 s.d. of the noise floor measured at nearby frequencies.

### Auditory brainstem responses and cochlear microphonics

ABRs and CMs were measured using routine techniques^70^. Briefly, the ABR potentials were measured from subcutanteous needle electrodes positioned near the left side neck muscles and at the vertex of the head, with a ground electrode placed in the left rear leg. A bioamplifier (DP-311, Warner Instruments, Hamden, CT, USA) was used to amplify the signal 10,000 times. Clicks were created by creating a step pulse of 3 ms duration. To calibrate the stimulus intensity, we measured the click emitted from the speaker with the microphone and determined the root-mean-square value of the sound during the time of the stimulus. During the experiments, the click intensity level was raised in 10 dB steps from 10 to 80 dB SPL. At each sound level, 260 responses were sampled and averaged. The peak-to-peak value of the ABR was measured. Thresholds were defined to be when the peak-to-peak value was 5x higher than the mean noise floor, which was measured during the last 20 ms of the repetition time, after the ABR response was completed.

The CM was measured using the same recording electrodes and bioamplifier. The stimuli were 20 ms 2.75 kHz tones with intensities ranging from 30 to 80 dB SPL. The CM magnitude was determined by Fourier transform (FFT).

### Histology

After euthanasia, the chicken basilar papillae were dissected out from the temporal bone and fixed in fixative mixture (25% glutaraldehyde and 4% paraformaldehyde in 0.1 M phosphate buffer) overnight at 4°. The tissue was then decalcified in 0.12 M EDTA in 0.1 M PB for 3 days. To view the gross anatomy of the inner ear organs, microdissection was performed to remove the surrounding otic capsule bone and images were taken through a Leica dissection microscope. To perform frozen section analysis, the basilar papilla was embedded in optimal cutting temperature (OCT) compound, frozen and cut in 10 μm sections. Unstained images were acquired using light microscopy with a Zeiss LSM5 Pascal system 227 and a 5X/0.5 EC Plan-NEOFLUAR ∞/0.17 objective.

### Data availability

All relevant data are available from the authors upon request.

## Additional information

**How to cite this article:** Xia, A. *et al*. Hair cell force generation does not amplify or tune vibrations within the chicken basilar papilla. *Nat. Commun.* **7,** 13133 doi: 10.1038/ncomms13133 (2016).

## Supplementary Material

Supplementary Movie 1 3D image stack of the apical half of the chicken basilar papilla. This stack was collected by taking cross sections across the width and serially moving the laser from the base to the apex. We were able to start collecting partial images ~40% along the length, starting from the base. We then scanned apically to the 95% region, just proximal to the lagena.

Supplementary Movie 2 Calculated traveling wave along the chicken basilar papilla in response to a 250 Hz tone presented at 50 dB SPL. The vibratory motion has been magnified 2000x. The movie was created by fitting a 3D mesh to match the outline of the auditory epithelium traced from the image stack presented in Supplementary Movie 1. The mesh was animated by transposing vibratory data that was measured at the 75% location along the length of the basilar papilla according to the formula for the tonotopic map in equation (1).

Supplementary Movie 3 Calculated traveling wave along the chicken basilar papilla in response to a 500 Hz tone presented at 50 dB SPL. This movie was created in the same way as Supplementary Movie 2.

Supplementary Movie 4 Calculated traveling wave along the chicken basilar papilla in response to a 1000 Hz tone presented at 50 dB SPL. This movie was created in the same way as Supplementary Movie 2.
